# Scattering Removal for Finger-Vein Image Restoration

**DOI:** 10.3390/s120303627

**Published:** 2012-03-15

**Authors:** Jinfeng Yang, Ben Zhang, Yihua Shi

**Affiliations:** Tianjin Key Lab for Advanced Signal Processing, Civil Aviation University of China, P.O. Box 9, Tianjin 300300, China; E-Mails: comm.benzhang@gmail.com (B.Z.); yh-shi@cauc.edu.cn (Y.S.)

**Keywords:** image restoration, finger-vein, scattering removal, optical model

## Abstract

Finger-vein recognition has received increased attention recently. However, the finger-vein images are always captured in poor quality. This certainly makes finger-vein feature representation unreliable, and further impairs the accuracy of finger-vein recognition. In this paper, we first give an analysis of the intrinsic factors causing finger-vein image degradation, and then propose a simple but effective image restoration method based on scattering removal. To give a proper description of finger-vein image degradation, a biological optical model (BOM) specific to finger-vein imaging is proposed according to the principle of light propagation in biological tissues. Based on BOM, the light scattering component is sensibly estimated and properly removed for finger-vein image restoration. Finally, experimental results demonstrate that the proposed method is powerful in enhancing the finger-vein image contrast and in improving the finger-vein image matching accuracy.

## 1. Introduction

Finger-vein recognition, as a highly secure and convenient technique of personal identification, has been attracted much attention for years. In contrast to conventional appearance-based biometric traits such as face, fingerprint and palmprint, finger-vein patterns are hidden beneath the human skin and unnoticeable without the help of some specific viewing or imaging devices. This makes finger-vein trait resistant to steal or forgery, and thereby highly reliable for identity authentication. However, in practical scenario, the inherent advantage of finger-vein can not always be made effectively for finger-vein recognition due to the low contrast of finger-vein images. Therefore, to exploit the genuine characteristics in finger-vein images, the visibility of finger-vein patterns should be improved reliably.

**Figure 1.**
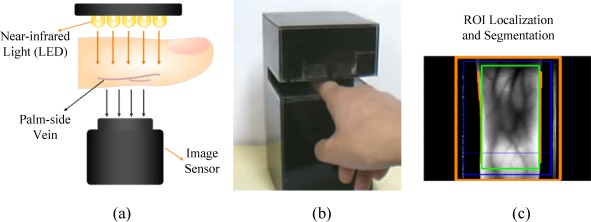
Finger-vein image acquisition system. (**a**) NIR light transillumination. (**b**) A homemade finger-vein imaging device. (**c**) ROI extraction proposed in [4].

**Figure 2.**
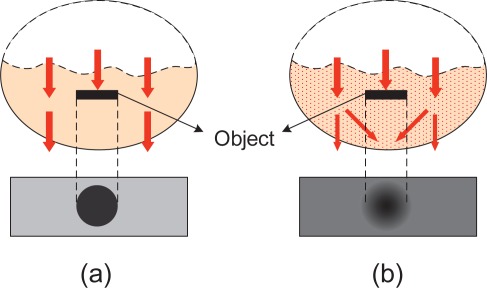
Image contrast reduction due to light scattering. (**a**) A real shadow as no light scattering. (**b**) A degraded shadow as light scattering.

Generally, in order to visualize finger-vein vessels inside the finger tissues, the near infrared (NIR) transillumination is often adopted in imaging devices, as shown in Figure 1(a,b). As the hemoglobin in blood vessels absorbs more NIR radiation than other substances in finger tissues [1], the intensity distribution of transmitted NIR rays vary spatially. Vein vessels cast darker “shadows” on imaging plane while other tissues present a brighter background, as shown in Figure 1(c). Since the biological tissues can be viewed as a kind of highly heterogeneous optical medium, multiple light scattering predominates in lights that penetrate through a biological tissue layer [2]. Thus, the quality of finger-vein images is always poor because the scattering effects can greatly reduce the contrast between the venous and non-venous regions [3]. The basic concept of image degradation due to light scattering is illustrated in Figure 2. If no light scattering is generated in optical medium, a real shadow of an object must appear on the imaging plane, as shown in Figure 2(a). However, the object shadow always is blurred to a certain extent since light scattering is inevitable in real situations, as shown in Figure 2(b).

Traditionally, for reliable vein-based recognition, many image enhancement methods have been proposed to improve the quality of vein images. Histogram equalization based algorithms were used to enhance the contrast between the venous and background regions in [5,6]. Considering the variations of vein-coursing directions, Yang *et al*. [7–10] used different oriented filtering strategies to highlight the finger-vein texture. Wang *et al*. [11] combined the fuzzy and the retinex theory to enhance the near-infrared vein images. Pi *et al*. [12] used edge-preserving filter and elliptic high-pass filter together to denoise and enhance some small blurred finger veins. Gao *et al*. [13] combined the traditional high frequency emphasis filtering algorithm and the histogram equalization to sharpen the image contrast. Oh *et al.* [14] proposed a homomorphic filter incorporating morphological subband decomposition to enhance the dark blood vessels. Although these methods can respectively enhance vein images to some extent, their performances were considerably undesirable in practice since they all did not treat of the key issue of light scattering in degrading finger-vein images.

Strong scattering occurring in the biological tissue during vein imaging is the main reason causing contrast deterioration in finger-vein images [15]. Therefore, for reliable finger-vein image contrast improvement, this paper aims to find a proper way of scattering removal according to tissue optics, especially skin optics.

In computer vision, scattering removal has been a hot topic for reducing the atmospheric scattering effects on the images of outdoor scenes [16–20]. This technique often is termed as dehazing or de-weather, which is based on a physical model that describes the formation of hazing image. Inspired by image dehazing, we here propose an optical-model-based scattering removal algorithm for finger-vein image enhancement. The proposed optical model allows for the light propagation in finger-skin layer such that it is powerful in describing the effects of skin scattering on finger-vein images.

In the following sections, a brief description of image dehazing model is firstly presented, and then the optical model used in this paper is derived after discussing the difference and relationship between our model and image dehazing model. In Section 3, the steps of scattering removal algorithm are detailed. For finger-vein image matching, Phase-Only-Correlation measure is used in Section 4. The experimental results are reported in Section 5. Finally, in Section 6, we give some conclusions.

## 2. The Optical Model

The physical model widely used to image dehazing, also named Koschmieder model, is expressed as [21]

(1) $$I_{d}=e^{-\mathrm{Kd}}I_{0}+(1-e^{-\mathrm{Kd}})I_{\infty }.$$

This model provides a very simple and elegant description for two main effects of atmospheric scattering on the observed intensity *I_d_* of an object at a distance *d* in a hazing or foggy day. Here, the intensity at close range (distance *d* = 0) *I*_0_ is called the intrinsic intensity of the object, *I_∞_* is the intensity of environmental illumination (equivalent to an object at infinite distance), which is generally assumed to be globally constant, and *K* is the extinction coefficient of the atmosphere.

**Figure 3.**
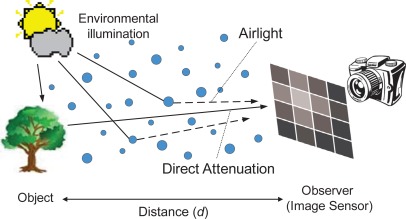
Effects of atmospheric scattering.

As illustrated in Figure 3, the first effect of atmospheric scattering is called direct attenuation, and can be described by Beer–Lambert law, which results in an exponential attenuation of object intensity with the transmission distance through scattering media, i.e., the first term *e*^−*Kd*^*I*_0_ on the right side of Equation (1). The second effect, referred to as airlight in Koschmieder theory of horizontal visibility, is caused by the suspended particles in haze or fog that scatter the environmental illumination toward the observer. The airlight acts as an additional radiation superimposed on the image of the object, whose intensity is related to the environmental illumination *I_∞_* and increases with pathlength *d* from the observer to the object, as described by the term (1 − *e*^−^*^Kd^*)*I_∞_*.

It is noticeable that, despite having not taken multiple scattering into account, the Koschmieder model is practicable for vision applications. In atmosphere, the distances between particles are usually large enough so that the particles can be viewed as independent scatterers, whose scattered intensities do not significantly interfere with each other, and thus the effect of multiple scattering is negligible [22]. Whereas, in the case of biological tissue, light propagation suffers a more complex process due to the complexity of tissue structure. Particularly, the scattering particles in biological tissue are so dense that the interaction of scattered intensities between neighboring particles cannot be ignored [23]. Hence, multiple scattering is said to be prevalent in the biological optical medium.

**Figure 4.**
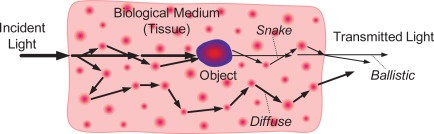
Light propagation through biological tissue.

From the biophotonic point of view, as the light propagates through a tissue, the transmitted light is composed of three components—the ballistic, the snake, and the diffuse photons [24], as shown in Figure 4. Ballistic photons travel a straight, undeviated path in the medium. Snake photons experience some slight scattering events, but still propagate in the forward or near-forward direction. Diffuse photons undergo multiple scattering and emerge from random directions. Obviously, in transillumination imaging of objects embedded in the biological tissue, the ballistic photons with propagation direction preservation can form sharp shadows of objects on the imaging plane, whereas the multiple scattered diffuse photons can inevitably reduce the contrast of the shadows as well as giving rise to the unwanted, incoherent imaging background [25]. That is to say, the multiple scattering is the most unfavorable factor that contributes to diffuse photons and further leads to image blurring in optical transillumination imaging.

**Figure 5.**
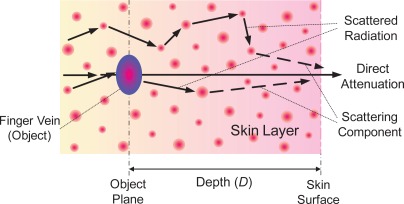
Simplified skin scattering model.

Based on the preceding analysis of image dehazing model, and associated with the knowledge of light propagation through biological tissue, we propose a simplified skin scattering model to characterize the effects of skin scattering on finger-vein imaging, as shown in Figure 5.

**Figure 6.**
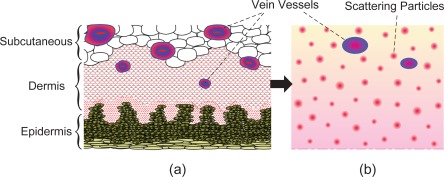
Skin layer modeling. (**a**) Cross-sectional view of human skin. (**b**) Simplified model of finger palm-side skin layer.

Before presenting the mathematical expression, there are several points with respect to the optical model should be stated:

1. In a real finger-vein imaging system, the objects to be visualized are palm-side vein vessels which are mostly interspersed within the inner layer of the finger skin (see Figure 6(a)). So, for the sake of simplicity, only the skin layer is considered as a reference optical medium regardless of the atmosphere between skin surface and camera, whose scattering effect is very small and negligible here.
2. Human skin is known to be an inhomogeneous, multilayered tissue containing epidermis, dermis and subcutaneous layer, as shown in Figure 6(a). But at the molecular level, skin tissues are composed of a limited number of basic molecular species, and these molecules are composed of optically similar chemical units [2]. Moreover, the ensemble of light-skin interaction homogenizes the optical behavior of biological structures. Thus, the skin can be viewed as a medium with a random but homogeneous distribution of scattering particles over its thickness [26], as shown in Figure 6(b), and then the scattering coefficient of the skin tissue here can be assumed to be constant.
3. Different from the image dehazing techniques, we need not consider the effect of environmental illumination as well as the airlight indeed. Nevertheless, due to light interaction occurs among biological scatterers, the scattered radiation from both the object and the background will be partially re-scattered towards the observer, which amounts to environmental illumination for finger-vein imaging.

**Figure 7.**
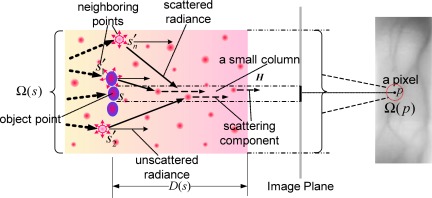
Schematic representation of the effect of scattered radiation.

In view of these points, the radiant intensity observed at skin surface corresponding to the object with a certain depth in the skin can be simply decomposed into the direct attenuation component and the scattering component, as shown in Figure 5. The former, representing the effect of ballistic photons, is a reduction of the original radiation over the traversing medium, which obeys the Beer–Lambert law, while the latter represents the effect of snake and diffuse photons, namely a proportion of scattered radiation enters into the direction of observer and interferes with the direct radiation of object, whose intensity increases with depth because a deeper object tends to suffer more influence of the scattered radiation. Accordingly, in a similar way of Koschmieder model, the proposed biological optical model (BOM) is defined as

(2) $$I(p)=e^{-\mu D(s)}I_{0}(s)+(1-e^{-\mu D(s)})I_{r}(s),$$

where *s* represents an original source, *p* is the observation of *s* on the imaging plane, *μ* denotes the extinction coefficient of the skin tissue (assumed to be constant here). So, *I*_0_(*s*) still represents the intrinsic intensity of the object, that is veins, to be visualized, *I_r_*(*s*) denotes the intensity of scattered radiation, and *I*(*p*) is the observation of the vein object on the image plane. A key point needs to be noted that, different from the environmental illumination in atmosphere, *I_r_*(*s*) varies spatially because its value is associated to the intensities of the imaging background. Figure 7 schematically illustrates the effect of scattered radiation for intuitively understanding the relation between the proposed skin scattering model and a finger-vein image.

Assume that *H* denotes a small column in the skin tissue corresponding to a beam from the object point *s* to a point *p* on the image plane (each pixel corresponds to a small column), then the neighbor points (*s^′^_i_* ,*i* = 1, 2, ⋯ ,*n*) around *s* can be viewed as the local background radiation sources, which would emit radiation into the column *H* and produce the scattering component of the transmitted radiation along *H*. Let the original intensity of a neighbor point *s^′^_i_* be *I*_0_(*s^′^_i_*), then the direct transmitted radiation of this point, that is the unscattered radiation, should be *e*^−^*^μD^*^(^*^s′^_i_*^)^*I*_0_(*s^′^_i_*). So, according to the energy conservation principle, the scattered radiation of this point should be (1 − *e*^−^*^μD^*^(^*^s′^_i_*^)^)*I*_0_(*s^′^_i_*), where *D*(*s^′^_i_*) is the depth of point *s^′^_i_* in the skin layer. Thus, we can obtain the scattered radiation *I_r_*(*s*) in *H*. Since the scattering directions are random, *I_r_*(*s*) here is considered as an average of total radiation from overall neighbor points and rewritten as

(3) $$I_{r}(s)=\frac{1}{Z_{\Omega (s)}}\sum_{s_{i}^{\prime }\in \Omega (s)}(1-e^{-\mu D(s_{i}^{\prime })})I_{0}(s_{i}^{\prime }),$$

where Ω(*s*) denotes the 2D neighborhood centered at point *s*, and *Z*_Ω_ indicates the number of points in Ω(*s*).

Given *I_r_*(*s*), and *D*(*s*), we can obtain *I*_0_(*s*) which represents the intrinsic intensity of a finger-vein image without scattering corruption. However, solving *I*_0_(*s*) from a single observed image *I*(*p*) with Equation (2) is a very ill-posed problem.

## 3. Scattering Removal Algorithm

The values of *I_r_*(*s*), *μ* and *D*(*s*) can not be evaluated accurately since the light scattering phenomenon in tissues is very complex. Not only is the extinct coefficient *μ* of human skin tissue inconsistent, but the thickness *D*(*s*) also varies with different individuals. Hence, referring to the image dehazing technique, we here introduce

(4) $$V(s)=(1-e^{-\mu D(s)})I_{r}(s).$$

*V* (*s*) can be regarded as the scattering component. Moreover, let *T* (*s*) = *e*^−^*^μD^*^(^*^s^*^)^ be the transmission map, we can obtain

(5) $$T(s)=1-\frac{V(s)}{I_{r}(s)}.$$

*T* (*s*) describes the relative portion of light radiation surviving through a medium. Thus, the optical model can be rewritten as

(6) $$I(p)=T(s)I_{0}(s)+V(s).$$

Instead of directly computing *I*_0_(*s*), we first estimate the scattering component *V* (*s*), and then estimate the intensity of scattered radiation *I_r_*(*s*). Thus, the restored image *I*_0_(*s*) can be obtained based on Equations (5) and (6).

### 3.1. Scattering Component Estimation

Unlike the regularized solution of scattering component estimation described in [17], *V* (*s*) here varies locally and spatially on finger-vein imaging plane due to the heterogeneousness of the human skin tissue. Hence, three practical constraints should be introduced for *V* (*s*) and *I_r_*(*s*) estimation: (1) For each point *s*, the intensity *V* (*s*) is positive and cannot be higher than the finally observed intensity *I*(*p*), that is, 0 *≤ V* (*s*) *≤ I*(*p*); (2) *V* (*s*) is smooth except the edges of venous regions since the points in Ω(*s*) approximate to be same in depth; (3) *I_r_*(*s*) tends to be constant in Ω(*s*) and *V* (*s*) *≤ I_r_*(*s*) *≤ I*(*p*). Based on these constraints, to estimate *V* (*s*), a fast algorithm described in [19] is modified as

(7) $$V(s)=1-\text{max}(\text{min}(w_{1}B(s),\;\hat{I}(p)),\;0),$$

where *B*(*s*) = *A*(*p*) − *median*_Ω(_*_p_*_)_(*|Î*(*p*) − *A*(*p*)*|*), *A*(*p*) = *median*_Ω(_*_p_*_)_(*Î*(*p*)), Ω(*p*) denotes the 2D neighborhood centered at point *p*, *w*_1_ (*∈* [0, 1]) is a factor controlling the strength of the estimated scattering component, and *Î*(*p*) is the negative version of *I*(*p*). In *Î*(*p*), the venous regions become bright and can be viewed as fluorescent sources emitting light in transcutaneous manner, which is beneficial for modeling the light scattering component.

### 3.2. Scattering Radiation Estimation

To obtain the transmission map *T* (*s*), we should compute *I_r_*(*s*). Intuitively, we can obtain *I_r_*(*s*) via Equation (3) directly. However, it is a difficult task since the intrinsic intensity *I*_0_(*s^′^_i_*) is unavailable in practice. Hence, considering the physical meaning that the scattered radiation *I_r_*(*s*) depends on the interaction among neighbor points in Ω(*s*), we here simply use a local statistic of Ω(*p*) to represent *I_r_*(*s*), that is,

(8) $$I_{r}(s)=\frac{w_{2}}{Z_{\Omega (p)}}\sum_{i=1}^{Z_{\Omega (p)}}I(p_{i}),$$

where *p_i_ ∈* Ω(*p*), *Z*_Ω(_*_p_*_)_ indicates the number of points in Ω(*p*), and *w*_2_ (*∈* [0, 1]) is a factor for making the constraint *V* (*s*) *≤ I_r_*(*s*) *≤ I*(*p*) satisfying. So, based on Equation (5), we can estimate *T* (*s*) accordingly.

### 3.3. Finger-vein Image Restoration

Given the estimations of *V* (*s*) and *T* (*s*), we can approximately restore an original finger-vein image with scattering removal. That is, by solving Equation (6) with respect to *I*_0_(*s*), we can obtain

(9) $$I_{0}(s)=\frac{I(p)-V(s)}{T(s)}.$$

Thus, computing *I*_0_(*s*) pixelwise using Equation (9) can generate an image *I*_0_(*x, y*) automatically and effectively. Here, *I*_0_(*x, y*) represents the restored finger-vein image which appears free of multiple light scattering.

## 4. Finger-vein Image Matching

In this section, the Phase-Only-Correlation (POC) measure proposed in [27] is simply used for handling the finger-vein matching problem based on the restored finger-vein images. Assume that *I*_0_*i*__ (*x, y*) and *I*_0_*j*__ (*x, y*) are two restored images, and *F_i_*(*u, v*) and *F_j_*(*u, v*) represent their 2D DFT, respectively, according to the property of Fourier transform, that is,

(10) $$I_{0_{i}}\;(x,y)\circ I_{0_{j}}\;(x,y)\Leftrightarrow F_{i}(u,v)\bar{F_{j}\;(u,v),}$$

where “ ○ ” denotes a 2D correlation operator, we can compute the cross phase spectrum as

(11) $$R(u,v)=\frac{F_{i}(u,v)\bar{F_{j}(u,v)}}{\left\|F_{i}(u,v)\bar{F_{j}(u,v)}\right\|}=e^{\hat{j}\theta (u,v)}.$$

**Figure 8.**
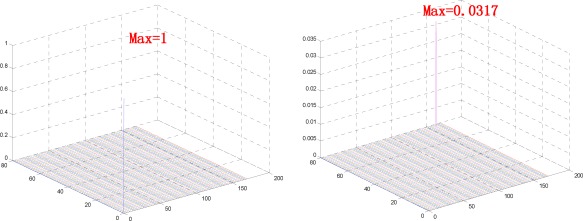
POC measure. Left: *r*(*x, y*) of two same finger-vein images. Right: *r*(*x, y*) of two finger-vein images from different classes.

Let *r*(*x, y*) = IDFT(*R*(*u, v*)), thus, *r*(*x, y*) is called a POC measure. The POC measure has a sharp peak when two restored finger-vein images are similar, whereas it will be near zero for those from different classes, as shown in Figure 8. Moreover, POC measure is insensitive to image shifts and noises in practice.

## 5. Experimental Results

In this section, the used finger-vein images are captured by a homemade transillumination imaging system with a 760 nm NIR LED array source, and then extracted from raw images by the ROI localization and segmentation method proposed in [4]. The finger-vein image database contains 700 individual finger-vein images from 70 individuals. Each individual contributes 10 forefinger-vein images of the right hand. All finger-vein images are 8-bit gray images with a resolution of 320 × 240.

### 5.1. Finger-vein Image Restoration

**Figure 9.**
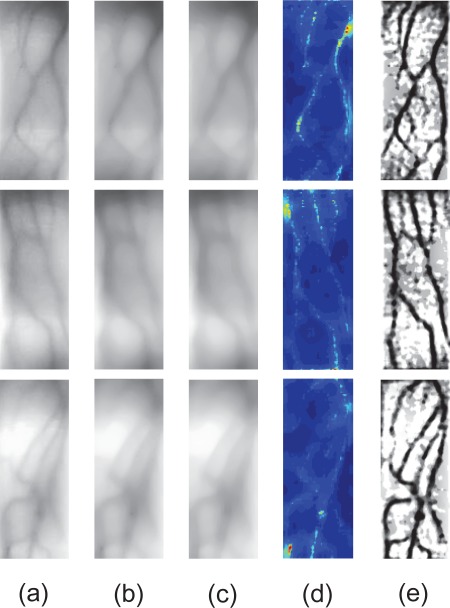
Scattering removal experiments. (**a**) Some captured finger-vein images *I*(*x, y*). (**b**) The estimated scattering components *V* (*x, y*). (**c**) The estimated scattering radiations *I_r_*(*x, y*). (**d**) The estimated transmission maps *T* (*x, y*). (**e**) The restored images *I*_0_(*x, y*).

Here, some captured finger-vein image samples are collected to demonstrate the validity of the proposed method in finger-vein image restoration. Figure 9 shows some examples of the estimated *V* (*x, y*), *I_r_*(*x, y*), *T* (*x, y*) and restored finger-vein images *I*_0_(*x, y*). After scattering removal, the contrast of finger-vein images is improved significantly, and the vein networks present in the restored images can be clearly distinguished from the background. This shows that the proposed optical model allowing for the effects of light scattering in skin layer, particularly the multiple scattering, is desirable for describing the mechanism of finger-vein image degradation.

Nevertheless, the proposed method is somewhat sensitive to image noises, as shown in Figure 9(e). In fact, before lighting the palm-side veins, the NIR rays have been randomly diffused by finger dorsal tissues such as finger-back skin, bone, tendon, fatty tissue and so on. This inevitably gives rise to irregular shadows and noises in the captured finger-vein images, whereas the proposed optical model has not taken account of the effects of finger dorsal tissues except the palm-side skin. As a result, the spatial varied background noises are also strengthened when estimating the scattering components.

**Figure 10.**
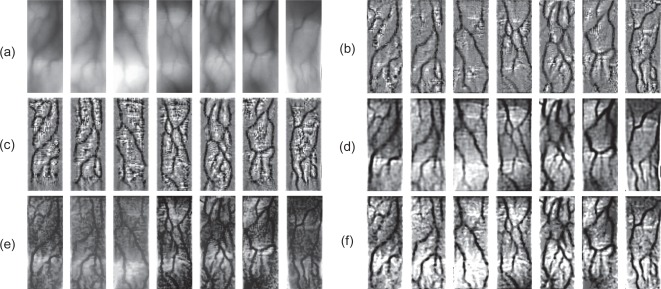
Comparisons with other methods. (**a**) Some captured finger-vein images. (**b**) The results from histogram template equalization (HTE) [5]. (**c**) The results from high frequency emphasis filtering (HFEF) [13]. (**d**) The results from circular Gabor filtering (CGF) [7]. (**e**) The results from image dehazing (ImD) [19]. (**f**) The results from the proposed method.

In Figure 10, we compare our method with several common approaches for finger-vein image enhancement. Additionally, we treat the degraded finger-vein images as hazing images, and directly use dehazing method to restore them regardless of the mismatch between the Koschmieder model and the proposed model. Here, a method proposed in [19] is adopted to implement finger-vein image “dehazing”, and the results are also shown in Figure 10.

In order to evaluate the performance of the proposed method in terms of contrast improvement for finger-vein image, the mean structural similarity index (MSSIM) [12] and the contrast improvement index (CII) [14] are used as two common evaluation criterions. We first randomly choose 50 individual finger-vein images from database as samples, and use these enhancement methods in Figure 10 to process the finger-vein image samples. Then, we obtain the average MSSIM and the average CII of every enhancement method.

In general, MSSIM is often used to measure the similarity between a processed image and a standard image with perfect quality (*i.e.*, a distortion-free image). The larger the value of MSSIM is, the better an image is improved. This makes a processed image more approximate to its standard quality. However, it is impossible for us to have standard or perfect finger-vein images since the captured images all are degraded due to light scattering. Therefore, we regard the degraded finger-vein images as standard references. Thus, the more the dissimilarity between a processed finger-vein image and its original version is, the better the finger-vein is improved. That is, the lower the value of MSSIM is, the better the quality of a restored image is. CII is often used to measure the improvement of contrast between a processed image and its original version, and the larger the value of CII is, the better the contrast of an improved image is.

Hence, the quality and the visibility of restored finger-vein images can be quantitatively evaluated using MSSIM and CII. In Table 1, we list the two values corresponding to different finger-vein enhancement methods. From Table 1, we can clearly see that the proposed method provides the lowest MSSIM value and the highest CII value. This means the proposed method has better performance in finger-vein image enhancement.

## 5.2. Finger-Vein Image Matching

**Figure 11.**
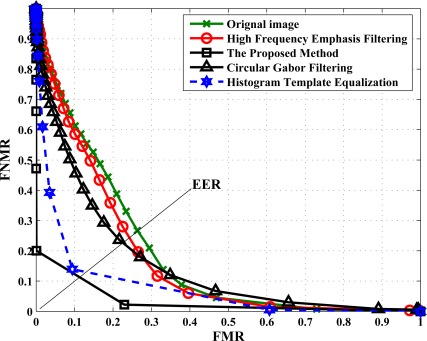
ROC curves of different finger-vein enhancement results.

For finger-vein matching on this database, the number of genuine attempts is 3,150 $\left(70C_{10}^{2}\right)$, and the number of impostor attempts is 241,500 $\left(10\times 10C_{70}^{2}\right)$. By respectively using the original images, HTE-based images, HFEF-based images, CGF-based images, ImD-based images and the proposed restored images for finger-vein matching under POC (Phase-Only-Correlation) measure, the ROC (receiver operating characteristic) curves are plotted in Figure 11, where false non-match rates (FNMR) and false match rates (FMR) are shown in the same plot at different thresholds on the POC matching score, and EER (equal error rate) is the error rate where FNMR and FMR are equal.

From Figure 11, we can clearly see that the proposed method has the best performance of ROC curves and makes the lowest EER. This indicates that the finger-vein images with scattering removal are more discriminative in inter-class. Hence, the proposed method is desirable for improving the accuracy of finger-vein image matching in practice.

## 6. Conclusions

In this paper, a scattering removal method was introduced for finger-vein image restoration. The proposed method was based on a biological optical model which reasonably described the effects of skin scattering. In this model, the degradation of finger-vein images was viewed as a joint function of the direct light attenuation and multiple light scattering. By properly estimating the scattering components and transmission maps, the proposed method could effectively remove the effects of skin scattering effects from finger-vein images to obtain the restored results. The comparative experiments and quantitative evaluations demonstrated that the proposed method could provide better results compared to the common methods for finger-vein image enhancement and recognition.

Indeed, the proposed method also had its own drawbacks. First, the simplified model in our work did not take into account of the effects of background tissues, which made the proposed method somewhat sensitive to image noises while enhancing the vein patterns. Besides, the rough estimations of the scattering components as well as the scattered radiations could also decrease the performance of the proposed method to some extent. All these shortcomings will be of our further improvement in future work.

## Figures and Tables

**Table 1.** Quantitative evaluation of different enhancement methods.

Methods	Average MSSIM	Average CII
The captured images	1	1
Histogram Template Equalization (HTE)	0.4076	4.4941
High Frequency Emphasis Filtering (HFEF)	0.4239	3.7571
Circular Gabor Filtering (CGF)	0.4141	3.7386
Image Dehazing (ImD)	0.4932	3.3967
The Proposed Method	0.3358	4.6210
